# Coming of Age: Facing Growing Pains in Structural Heart Interventions With Innovative Imaging Solutions

**DOI:** 10.1016/j.jscai.2025.104016

**Published:** 2025-10-07

**Authors:** Megan Coylewright

**Affiliations:** Heart and Vascular Center, Essentia Health, Duluth, Minnesota

**Keywords:** intracardiac echocardiography, left atrial appendage closure, structural heart, tricuspid repair

Evidence that transcatheter structural heart disease therapies have come of age is in front of us: the focus of interventional cardiology is now on capacity and infrastructure over simply growth. The demand for mature therapies—including transcatheter aortic valve replacement, left atrial appendage occlusion (LAAO), and transcatheter edge-to-edge repair—may soon outstrip our human resources in many hospitals in the United States and thus requires thoughtful implementation of innovations in efficiency.

In this context, the Society for Cardiovascular Angiography & Interventions (SCAI) provides a position statement on the use of intracardiac echocardiography (ICE) to guide structural heart interventions.^1^ This follows the recent 2025 SCAI/Heart Rhythm Society Clinical Practice Guidelines on Transcatheter LAAO and accompanying technical review, distinct documents involving review of the literature to describe the safety and efficacy of ICE for LAAO^2^^,^^3^; however, the current position statement provides a more comprehensive review of ICE for all structural heart interventions and provides some step-by-step guidance for interventional cardiologists (ICs) as their practices evolve to include this critical imaging modality. Structural heart ICs must consider several aspects regarding both why and how to consider integrating ICE into their practice.

## Adjunctive visualization

Limitations exist in imaging from transesophageal echocardiography (TEE) based on unique patient anatomy in addition to the location of structures of interest (ie, anterior location of tricuspid valve). For some patients, TEE can have higher complications, and case selection remains important.^4^ ICE can provide a closer, more nuanced view to cardiac structures needed to confirm positioning and safe implantation of devices.

The position statement succinctly reviews the advantages and limitations of ICE for visualization in structural heart procedures, noting the rapid development of new imaging probes and software to improve images. While early experience with manipulation of ICE probes led to a low signal of increased pericardial effusion, the tools continue to become smaller and more flexible, and operator skill sets and awareness as volumes of cases increase drive safety.^5^^,^^6^

## Expanding skill sets

Many ICs are less well versed in cardiac imaging compared with their highly refined procedural acumen. For many years, ICs have relied on their imaging colleagues not only to obtain images during structural heart procedures but also to provide nuanced interpretation and procedural guidance. For ICs to potentially transition to a dual role of implanter and imager requires formal and detailed training. This position statement is a small step to describe the differences and similarities for ICE across therapeutic areas and highlights that becoming a cardiac imager cannot be done ad hoc.

The fact that specialized postfellowship training via literature review, procedural videos, courses, and proctoring is supported by this position statement highlights the unique aspects of integrating a new imaging modality into practice. For some interventionalists, imaging remains a softer skill set that proceduralists may feel they can learn on their own. Evidence from the very slow uptake of ICE to guide LAAO due to difficulty crossing the interatrial septum, concern about navigating the probe in the left atrium due to risk of pericardial effusion, and uncertainty in interpretation of a new set of images magnifies the importance of training to increase the speed of the learning curve and adoption while ensuring safe best practices.

## Focus on both improved access and safety

ICE has the potential to increase efficiency in our catheterization laboratories at a time when greater throughput is desperately needed to address barriers to access for many patients. Our patients face very real threats of bleeding, stroke, heart failure, and death while on a waiting list for procedures such as transcatheter valve repair and replacement or LAAO, yet efficiency without assured safety is not the answer. Training in the use of ICE and incorporation of the insights those images provide is essential.

As mentioned, difficulties with scheduling structural heart therapies exist due to availability of structural heart imagers for TEE and anesthesiology teams to provide general anesthesia, preferred with TEE. ICE can be deployed under conscious sedation, without the requirement for airway protection necessitating endotracheal intubation; however, as many ICs invoke as these conversations arise, efficiency cannot take precedence over safety. This is why training remains essential when adding new skill sets and tools to structural heart procedures and respect for imaging as a tool is needed.

## Importance of training and education

Training goes beyond the images, however, and extends to additional aspects of using ICE in patients under conscious sedation. As laid bare from the recent US Food and Drug Administration Early Alert, left atrial procedures performed under conscious sedation, as with most ICE cases, carry risk if operators are unaware or do not pay close attention to unique aspects of the procedure.^7^ The US Food and Drug Administration alert, while named to focus on the WATCHMAN Access Sheath (Boston Scientific), is in reality an agnostic warning for any operator performing procedures in the low-pressure left atrium when patients are not under positive pressure ventilation. If patients are snoring or take a deep breath without the implanter assuring adequate volume loading and attention to minimizing any entry of air into open systems during procedure, rare yet catastrophic consequences can occur due to air embolism.

In this context, educational guidance from professional societies remains critical for patient safety and procedural effectiveness. Structural heart operators must acknowledge that our skill sets continue to go through seasons of coming of age, necessitating a humble approach to new modalities—and not just complex therapeutic devices but also our imaging tools. ICE will remain an important innovative solution to our current challenges of capacity and human resources. The SCAI Position Statement on use of ICE is an important first step, and ICs will benefit their patients by continuing to seek out educational and training opportunities to ensure safety and effectiveness as we address the growing pains of our own success.
